# Supplementary material for: Selection and Incorporation of siRNA Carrying Non-Viral Vector for Sustained Delivery from Gellan Gum Hydrogels
Source: Pharmaceutics. 2021 Sep 23;13(10):1546. doi: 10.3390/pharmaceutics13101546 (PMC8540443; doi:10.3390/pharmaceutics13101546)
Supplement: Supplementary file 1 [file pharmaceutics-13-01546-s001.zip › pharmaceutics-1367228-SM.pdf]

pharmaceutics

MDPI

Supplementary material

Selection and Incorporation of siRNA Carrying Non-Viral Vector for Sustained Delivery from Gellan Gum Hydrogels

Anastasios Nalbadis, Marie-Luise Trutschel, Henrike Lucas, Jana Luetzkendorf, Annette Meister and Karsten Mäder

Methods

S1.1 TEM

The negatively stained samples were prepared by spreading 5 µL of the dispersion (0.5 mg/mL) onto a Cu grid coated with a Formvar-film (PLANO, Wetzlar, Germany). After 1 min excess liquid was blotted off with filter paper and 5 µL of 1% aqueous uranyl acetate solution were placed onto the grid and drained off after 1 min. The dried specimens were examined with an EM 900 transmission electron microscope (Carl Zeiss Microscopy GmbH, Oberkochen, Germany). Micrographs were taken with a SSCCD SM-1k-120 camera (TRS, Moorenweis, Germany).

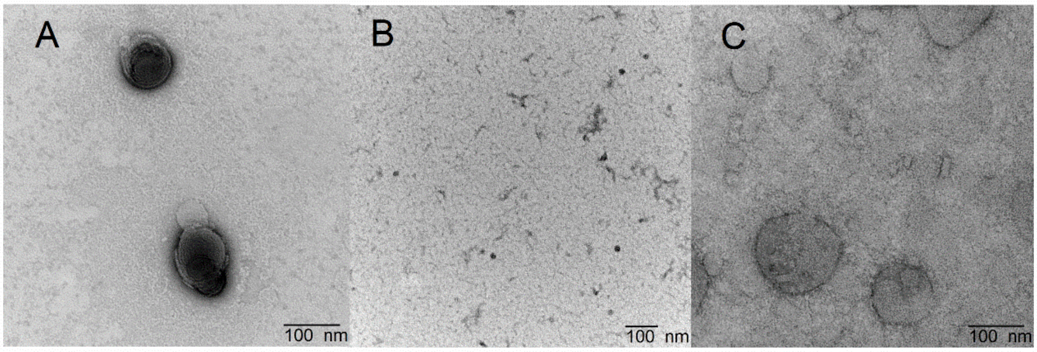

Figure S1. Transmission electron microscopy images of DL (**A**), CP (**B**), and SLC (**C**).
